# Gut-to-brain interoceptive pathways in emotion-related behaviors

**DOI:** 10.3389/fnsys.2026.1855213

**Published:** 2026-06-24

**Authors:** Tomofumi Otsuki, Takako Ichiki, Miho Terunuma

**Affiliations:** Division of Oral Biochemistry, Faculty of Dentistry, Graduate School of Medicine, Dentistry and Health Sciences, Niigata University, Niigata, Japan

**Keywords:** emotion, enteric nervous system, gut-brain axis, interoception, spinal afferents, vagal afferents

## Abstract

Emotion-related states encompass a wide range of affective and behavioral dimensions, including anxiety, fear, pleasure, and aversion, whereas motivation refers to internal drives that energize goal-directed behaviors such as feeding, reward seeking, or avoidance. These states are thought to arise from the brain’s integration of exteroceptive inputs, interoceptive signals reflecting bodily states, and prior experiences. Recent studies have increasingly highlighted the critical role of gut-to-brain communication, particularly vagal and other interoceptive pathways, in shaping emotion-related and motivational behaviors. In parallel, advances in genetic manipulation techniques have enabled the identification of neuronal circuits innervating the gastrointestinal tract and have provided new insights into their links with affective-like behavior. A better understanding of these mechanisms will be essential for elucidating how gastrointestinal-derived signals contribute to emotion-related states and how they are represented and processed in the brain. In this review, we discuss recent advances in gut-to-brain neural pathways, with a particular focus on vagal afferent and interoceptive mechanisms, while also considering spinal afferent pathways and ENS-related mechanisms that may contribute to emotion-related behavioral regulation.

## 1 Introduction

Afferent signals from the gastrointestinal system to the brain are increasingly recognized as important contributors to emotion-related and motivational states. Consistent with this view, patients with gastrointestinal disorders such as inflammatory bowel disease show a markedly increased prevalence of depression and anxiety disorders, suggesting that afferent signals associated with mechanical and chemical stimuli, as well as immune responses, may influence affective symptoms (Barberio et al., 2021; Bisgaard et al., 2022). Moreover, accumulating evidence has linked alterations in the gut microbiota to emotion-related symptoms and psychiatric disorders (McGuinness et al., 2022; Wang T. T. et al., 2026), and the therapeutic efficacy of vagus nerve stimulation (VNS) has been demonstrated in both clinical and preclinical settings. Gastrointestinal signals reach the brain through multiple routes, including vagal and spinal afferent pathways, as well as indirect signaling involving the enteric nervous system (ENS), immune mediators, and humoral factors (Wang et al., 2025). Nevertheless, direct causal evidence linking these pathways to emotion-related behavioral regulation remains limited.

In this review, we summarize rodent studies conducted under both physiological and pathological conditions, with a primary focus on vagal afferent and interoceptive mechanisms linking gastrointestinal signals to emotion-related behaviors. We also discuss spinal afferent pathways and ENS-related mechanisms where direct evidence is available, while acknowledging that these non-vagal routes remain less extensively characterized in the context of affective-like behavior. Because rodent behavioral assays do not directly measure subjective emotional experience, we use the terms “emotion-related behavior,” “affective-like behavior,” and “motivational behavior” according to the behavioral domain being examined. We highlight studies that have directly examined the relationship between internal organ-derived signals, especially those originating from the gastrointestinal tract, and behavioral regulation using denervation approaches such as vagotomy, electrical stimulation, pharmacological interventions, and genetic manipulations.

### 1.1 Vagal pathway

Visceral afferent signals carried by the vagus nerve are transmitted to the brainstem via sensory neurons in the nodose ganglion (NG). Classical experimental approaches, including vagotomy and subdiaphragmatic vagotomy (SDV), have been used to investigate vagal function. However, these manipulations affect not only afferent fibers but also, to some extent, efferent fibers. Studies employing VNS have additional limitations. First, conventional VNS does not selectively target gut-derived signals from specific organs. Second, it can activate both afferent and efferent fibers, making it difficult to attribute behavioral effects to a defined ascending interoceptive pathway. In contrast, targeted delivery of cholecystokinin-saporin (CCK-SAP) to the NG enables selective ablation of gastrointestinal vagal afferent pathways by inhibiting protein synthesis and inducing cell death in CCK-responsive neurons (Diepenbroek et al., 2017). More recently, advances in genetic techniques, including the use of transgenic mouse lines combined with adeno-associated virus (AAV)-mediated approaches, and single-cell RNA sequencing of vagal ganglia, have enabled precise manipulation of neural activity and the identification of distinct neuronal subtypes (Kupari et al., 2019; Bai et al., 2019; Ichiki et al., 2022). Collectively, these approaches permit selective targeting of organ-specific NG neurons and offer new opportunities to elucidate how gastrointestinal signals influence emotion-related and motivational behaviors. Studies examining anxiety-like and depression-like behaviors, fear extinction, reward, and motivation are summarized in Table 1.

**Table 1:** Summary of vagal pathway-related studies in emotion-related and motivational behaviors.

Anxiety-like behaviors										
A. Normal conditions					B. Pathological conditions					
Methods	Gain/Loss	Phenotype	Behavioral tests	Ref.	Models	Methods	Gain/Loss	Phenotype	Behavioral tests	Ref.
VNS	Gain (+)	Anxiety-like behavior ↓	EPM	Noble et al. (2019)	Social defeat stress	VNS	Gain (+)	Anxiety-like behavior ↓	EPM	Okonogi et al. (2024)
Gq-DREADDs (gastro-duodenal NG)	Gain (+)	Anxiety-like behavior ↑	OFT, EPM, and food neophobia test	Krieger et al. (2022)	PTSD model	VNS	Gain (+)	Anxiety-like behavior ↓	OFT, EPM, and NSF	Souza et al. (2020)
Gq-DREADDs (Oxtr^+^ NG)	Gain (+)	Vigilance-related behavior ↑	Indirect calorimetry/behavioral analysis	Scott et al. (2025)	DSS-induced colitis	SDV, CCK-SAP	Loss (−)	Anxiety-like behavior ↓	OFT, EPM, LDB and NSF	Chen et al. (2023)
SDA	Loss (−)	Anxiety-like behavior ↓	OFT, EPM, and food neophobia test	Klarer et al. (2014)	*B. longum*-treated colitis	SDV	Loss (−)	Anxiolytic effect abolished	Step-down test	Bercik et al. (2011)
CCK-SAP (NG)	Loss (−)	Anxiety-like behavior ↓	OFT, acoustic startle reflex test, and food neophobia test	Krieger et al. (2022)	0.1% iodoacetamide-induced FD	SDV	Loss (−)	Anxiety-like behavior ↓	OFT	Cordner et al. (2021)
Cervical vagotomy	Loss (−)	Anxiety-like behavior ↑	EPM	Okonogi et al. (2024)	*L. rhamnosus*-treated mice	SDV	Loss (−)	Anxiolytic effect abolished	EPM	Bravo et al. (2011)
					Transfer of gut microbiota from chronically stressed mice	SDV	Loss (−)	Anxiety-like behavior ↓	NSF	Siopi et al. (2023)
					High-fat diet-induced obesity	Caspase ablation (liver-innervating Avil^+^ NG neurons)	Loss (−)	Anxiety-like behavior ↓	OFT, EPM, and LDB	Hwang et al. (2025)

Depression-like behaviors										
C. Normal conditions					D. Pathological conditions					
Methods	Gain/Loss	Phenotype	Behavioral tests	Ref.	Models	Methods	Gain/Loss	Phenotype	Behavioral tests	Ref.
VNS	Gain (+)	Depression-like behavior ↓	FST	Krahl et al. (2004)	PTSD model	VNS	Gain (+)	Fear extinction ↑	Auditory fear conditioning	Souza et al. (2020)
VNS	Gain (+)	Depression-like behavior →	FST	Biggio et al. (2009)	PTSD model	VNS	Gain (+)	Fear extinction ↑	Auditory fear conditioning	Noble et al. (2017)
VNS	Gain (+)	Fear extinction ↑	Auditory fear conditioning	Noble et al. (2019)	PTSD model	VNS	Gain (+)	Fear extinction ↑	Auditory fear conditioning	Souza et al. (2021)
VNS	Gain (+)	Fear extinction ↑	Auditory fear conditioning	Peña et al. (2013)	0.1% iodoacetamide-induced FD	SDV	Loss (−)	Depression-like behavior ↓	FST	Cordner et al. (2021)
VNS	Gain (+)	Fear extinction ↑	Auditory fear conditioning	Peña et al. (2014)	*L. rhamnosus*-treated mice	SDV	Loss (−)	Antidepressant-like effect abolished	FST	Bravo et al. (2011)
VNS	Gain (+)	Fear extinction ↑	Auditory fear conditioning	Alvarez-Dieppa et al. (2016)	Transfer of gut microbiota from chronically stressed mice	SDV	Loss (−)	Depression-like behavior ↓	FST, TST, and sucrose preference test	Siopi et al. (2023)
VNS	Gain (+)	Fear extinction ↑	Auditory fear conditioning	Noble et al. (2017)						
SDA	Loss (−)	Conditioned Fear extinction ↓	Auditory fear conditioning	Klarer et al. (2014)						

Motivational behaviors										
E. Normal conditions					F. Pathological conditions					
Methods	Gain/Loss	Phenotype	Behavioral tests	Ref.	Models	Methods	Gain/Loss	Phenotype	Behavioral tests	Ref.
Optogenetic stimulation of gut-innervating vagal sensory pathways	Gain (+)	Reward-related preference ↑	RTPP	Sahasrabudhe et al. (2024)	Amphetamine or cocaine administration	SDV	Loss (−)	Drug-induced place preference ↓	CPP	Onimus et al. (2026)
Optogenetic stimulation of right NG neurons innervating the upper gut	Gain (+)	Reward-related preference ↑	Operant conditioning task and RTPP	Han et al. (2018)						
SDV	Loss (−)	Reward preference, Reward discrimination, and motivation ↓	T-maze test; operant conditioning task (lever pressing; FR and PR schedules)	Onimus et al. (2026)						

Avil, advillin; CCK-SAP, cholecystokinin-saporin; Chat, choline acetyltransferase; ChR2, channelrhodopsin-2; CPP, conditioned place preference; DREADDs, designer receptors exclusively activated by designer drugs; DSS, dextran sodium sulfate; ENS, enteric nervous system; EPM, elevated plus maze; FD, functional dyspepsia; FR, fixed ratio; FST, forced swim test; LDB, light–dark box; NG, nodose ganglion; NSF, novelty-suppressed feeding; OFT, open field test; Oxtr, oxytocin receptor; PR, progressive ratio; PTSD, post-traumatic stress disorder; RTPP, real-time place preference; SDV, subdiaphragmatic vagotomy; TST, tail suspension test; VNS, vagus nerve stimulation.

#### 1.1.1 Anxiety

Anxiety-like behaviors in rodents are commonly assessed as innate responses using behavioral assays such as the open field test (OFT), elevated plus maze (EPM), light–dark box (LDB), and food neophobia test, which assesses avoidance or reduced intake of novel food or solutions. Several studies have examined the role of vagal signaling in the regulation of anxiety-like behavior (Table 1). Rats treated with VNS exhibited increased time spent in the open arms of the EPM, indicating an anxiolytic-like effect (Noble et al., 2019). Likewise, rats subjected to SDV displayed anxiolytic-like behaviors in the OFT, EPM, and food neophobia test (Klarer et al., 2014). These rats also exhibited increased gamma-aminobutyric acid (GABA) levels in the ventral prefrontal cortex (vPFC) and decreased noradrenaline levels in the vPFC and nucleus accumbens (NAc). Similarly, rats receiving CCK-SAP injections into the NG exhibited anxiolytic-like behaviors in the OFT, acoustic startle reflex test, and food neophobia test in male rats, whereas in female rats, these effects were limited to the food neophobia test (Krieger et al., 2022). In the same study, transcriptomic analysis of neurons within the central nucleus of the amygdala (CeA), identified by transsynaptic tracing from the vagus nerve, revealed gene expression changes associated with anxiety disorders together with enhanced inhibitory synaptic transmission. Moreover, intra-CeA administration of the GABA_A_ receptor antagonist bicuculline abolished the anxiolytic-like behavior induced by CCK-SAP injection into the NG (Krieger et al., 2022). In contrast, cervical vagotomy induced anxiety-like behavior in the EPM, together with altered synchrony of neural oscillatory activity between the prefrontal cortex (PFC) and amygdala, resembling patterns observed in chronic stress-induced anxiety models (Okonogi et al., 2024). Together, these results suggest that vagal afferent signaling regulates anxiety-like behavior through polysynaptic circuits linking the gastrointestinal tract to limbic regions.

More direct evidence has emerged from studies using Designer Receptors Exclusively Activated by Designer Drugs (DREADD)-based chemogenetic approaches. Chemogenetic activation of NG neurons innervating the stomach and duodenum induced anxiety-like behavior in the OFT, EPM, and food neophobia test (Krieger et al., 2022). In addition, activation of oxytocin receptor-expressing NG neurons (NGOxtr) has been reported to induce vigilance-related behavioral responses together with marked hypometabolic changes, suggesting that distinct vagal sensory subtypes may regulate stress-related internal states (Scott et al., 2025).

The contribution of vagal pathways to anxiety-like behavior has also been reported in pathological conditions (Table 1). In a social defeat stress model, VNS restored EPM behavior toward baseline levels, indicating an anxiolytic effect (Okonogi et al., 2024). Similarly, VNS produced anxiolytic-like effects in the OFT, EPM, and novelty-suppressed feeding (NSF) test in a rat model of post-traumatic stress disorder (PTSD) (Souza et al., 2020). In dextran sodium sulfate (DSS)-induced colitis models, gastrointestinal vagal afferent signaling contributed to heightened anxiety-like behavior, and bilateral gastrointestinal vagal afferent ablation prevented the emergence of these behavioral abnormalities (Chen et al., 2023). Subsequent microbiome-based intervention studies further supported the involvement of vagal pathways in gut-brain communication. In particular, the anxiolytic effect of *Bifidobacterium longum* NCC3001 depended on intact vagal pathways (Bercik et al., 2011). Consistent with this, SDV also exerted anxiolytic-like effects in functional dyspepsia (FD) models (Cordner et al., 2021). In addition, the anxiolytic effect of *Lactobacillus rhamnosus* administration was abolished by SDV, further supporting the idea that intact vagal signaling is required for microbiota-dependent modulation of anxiety-like behavior (Bravo et al., 2011). Moreover, stress-induced alterations in the gut microbiota have been shown to require intact vagal signaling to influence affective behavior (Siopi et al., 2023). In addition, selective ablation of liver-innervating vagal sensory neurons induced anxiolytic-like behaviors in a high-fat diet-induced obesity model (Hwang et al., 2025).

Taken together, these findings indicate that vagal afferent signaling influences anxiety-like behavior in a context-dependent manner. Activation or disruption of gastrointestinal vagal afferents can produce different outcomes depending on physiological, inflammatory, metabolic, or microbiota-related states. These effects may involve anatomically and molecularly distinct NG neuron subtypes rather than a single uniform vagal pathway.

The divergent effects of SDV, CCK-SAP-mediated gastrointestinal vagal deafferentation, and cervical vagotomy may also reflect differences in anatomical specificity and affected fiber populations. SDV and CCK-SAP preferentially disrupt abdominal or gastrointestinal vagal afferents, whereas cervical vagotomy affects broader vagal afferent and efferent fibers from multiple organs. Species, sex, behavioral assays, and physiological or pathological conditions may further contribute to these contrasting outcomes. Thus, anxiety-like behaviors should be interpreted carefully when feeding, energy expenditure, pain, locomotor activity, or other physiological states may be altered.

#### 1.1.2 Depression

VNS has demonstrated therapeutic efficacy in patients with depression, and its clinical application continues to expand (Conway et al., 2025). However, preclinical studies directly examining the contribution of gastrointestinal-brain interactions to depression-like behavior remain relatively limited. Current evidence from animal studies is mixed. Whereas one study reported an antidepressant-like effect of VNS (Krahl et al., 2004), another found no significant effect in the forced swim test (FST), despite detecting VNS-induced neuronal plasticity in the hippocampus (Biggio et al., 2009) (Table 1). In an FD model, SDV reduced immobility time in the FST, indicating an antidepressant-like effect (Cordner et al., 2021) (Table 1). Moreover, administration of *Lactobacillus rhamnosus* produced an antidepressant-like effect and altered GABA_B1b_ and GABA_Aα2_ mRNA expression in multiple brain regions; both the behavioral and molecular effects were abolished by SDV (Bravo et al., 2011). Consistent with this, stress-induced alterations in the gut microbiota have also been shown to require intact vagal signaling to promote hippocampal plasticity changes and depressive-like behaviors (Siopi et al., 2023). These findings suggest that gastrointestinal signals transmitted through the vagus nerve may influence depression-like behavior and neuronal plasticity in the brain.

The mixed preclinical evidence for vagal regulation of depression-like behavior may reflect differences in stimulation parameters, VNS duration, behavioral assays, and disease models. Because conventional VNS is not specific to gastrointestinal afferent pathways, organ- and cell-type-specific manipulations will be needed to determine whether gut-derived vagal signals causally regulate depression-like behaviors.

#### 1.1.3 Fear

In rodents, fear-related behavior is commonly evaluated using Pavlovian fear conditioning paradigms, in which freezing responses to conditioned stimuli serve as a primary behavioral measure. Studies in rats have consistently shown that VNS facilitates the extinction of conditioned fear following auditory fear conditioning (Noble et al., 2019; Peña et al., 2013; Peña et al., 2014; Alvarez-Dieppa et al., 2016; Noble et al., 2017) (Table 1). This VNS-mediated effect was abolished by vagotomy (Klarer et al., 2014), supporting the involvement of vagal pathways. Although the underlying mechanisms remain to be fully elucidated, VNS appears to promote neuronal plasticity in brain regions critically involved in fear processing, including the basolateral amygdala (BLA) and medial prefrontal cortex (mPFC). Indeed, VNS has been reported to alter synaptic plasticity between the BLA and mPFC, and to modulate the expression of plasticity-related molecules such as activity-regulated cytoskeleton-associated protein (Arc), Ca^2+^/calmodulin-dependent protein kinase II, and GluN2B-containing NMDA receptors in the BLA (Peña et al., 2014; Alvarez-Dieppa et al., 2016). In rat models of PTSD, extinction of conditioned freezing responses was delayed relative to controls. However, when VNS was paired with extinction training, it reduced freezing behavior during training and decreased freezing responses during subsequent recall tests, indicating that VNS can restore impaired fear extinction (Souza et al., 2020; Noble et al., 2017; Souza et al., 2021) (Table 1). Taken together, these findings suggest that VNS promotes extinction of conditioned fear memory. Nevertheless, direct validation using genetic and other circuit-specific approaches remains limited, and the precise role of vagal signaling in fear regulation has yet to be fully defined.

#### 1.1.4 Reward, preference, and motivation

Recent studies have demonstrated that gastrointestinal vagal afferents also contribute to reward, preference, and motivation. Here, we use “motivation” to refer to internal drives that energize goal-directed behavior rather than to an emotional state itself. Optogenetic activation of gut-innervating vagal sensory pathways has been shown to increase time spent on the stimulated side in the real-time place preference (RTPP) test, indicating a role in reward-related preference (Sahasrabudhe et al., 2024) (Table 1). Likewise, optogenetic activation of right-sided NG neurons innervating the upper gut enhanced both self-stimulation behavior and place preference, as assessed by operant conditioning paradigms and RTPP. This manipulation was accompanied by increased dopamine signaling in the dorsal striatum, suggesting recruitment of positive reinforcement circuits (Han et al., 2018). By contrast, activation of left-sided NG neurons did not produce similar behavioral changes or alterations in dopamine signaling. These findings indicate that even NG neurons innervating the same organ can exhibit lateralized functional specialization. Consistent with this idea, SDV reduced preference for water and food and impaired reward discrimination in the T-maze test (Onimus et al., 2026). Although SDV did not affect operant learning itself, it reduced the number of trials completed under both fixed-ratio and progressive-ratio schedules, suggesting reduced motivation. A recent study further extended this concept by showing that post-ingestive sugar and fat signals recruit spatially distinct dorsal hippocampal neuronal ensembles through a vagal pathway, thereby regulating nutrient-specific preference, contextual memory, and motivation (Yang et al., 2025). SDV also suppressed drug-induced place preference behavior, further suggesting that gastrointestinal interoceptive signals contribute to both natural reward processing and addiction-related behavioral phenotypes (Onimus et al., 2026) (Table 1).

### 1.2 ENS and spinal afferent pathways

The ENS is an autonomous neural network that regulates gastrointestinal function independently of the brain. In addition to this intrinsic role, ENS-derived signals may influence gut-to-brain communication through vagal pathways, spinal afferent pathways mediated by dorsal root ganglion (DRG) neurons, epithelial intermediates, immune mediators, and humoral routes. The causal contribution of the ENS itself to emotion-related behavioral regulation remains poorly understood. Recent advances in single-cell RNA sequencing and genetic manipulation techniques have enabled detailed characterization of enteric neurons and glial cells, as well as precise recording and manipulation of their activity (Shi et al., 2025; Millett et al., 2025). Here, we highlight two studies using DSS-induced colitis model mice that directly examined the relationship between ENS activity and affective-like behaviors through genetic and pharmacological manipulation of enteric neurons and glial cells. These ENS-related findings are summarized in Table 2.

**Table 2:** Summary of ENS-related mechanisms in emotion-related behaviors.

Pain, aversion, and emotion-related behaviors										
A. Normal conditions					B. Pathological conditions					
Methods	Gain/Loss	Phenotype	Behavioral tests	Ref.	Models	Methods	Gain/Loss	Phenotype	Behavioral tests	Ref.
ChR2 (Chat^+^ ENS neurons in the distal colon)	Gain (+)	Pain-related behavior ↑, aversion →	Back-hunching behavior and step-down test	Wang Z. et al. (2026)	DSS-induced colitis	ChR2 (Chat^+^ ENS neurons in the distal colon)	Gain (+)	Aversion ↑	Step-down test	Wang Z. et al. (2026)
					DSS-induced colitis	DL-fluorocitrate barium salt (glial inhibitor)	Loss (−)	Anxiety-like behavior ↓, depression-like behavior ↓	OFT, EPM, and TST	Li et al. (2024)

Chat, choline acetyltransferase; ChR2, channelrhodopsin-2; DRG, dorsal root ganglion; DSS, dextran sodium sulfate; ENS, enteric nervous system; EPM, elevated plus maze; OFT, open field test; TST, tail suspension test.

Optogenetic activation of choline acetyltransferase-positive (Chat^+^) enteric neurons innervating the colon induced back-hunching behavior, a pain-related defensive response, via an ENS-DRG circuit, but did not elicit aversive behavior in the step-down assay under physiological conditions. In contrast, in DSS-treated mice, activation of Chat^+^ enteric neurons altered step-down assay performance, suggesting that inflammatory conditions unmask a contribution of these neurons to aversive behavior. These findings suggest that inflammation alters the functional properties of Chat^+^ enteric neurons, enabling them to contribute not only to pain-related defensive responses but also to aversive behavior (Wang Z. et al., 2026) (Table 2).

In parallel, another study using DSS-treated mice reported an increased number of GFAP-positive enteric glial cells, together with marked morphological alterations. Importantly, inhibition of enteric glial cell activity ameliorated both anxiety- and depression-like behaviors (Li et al., 2024) (Table 2). Collectively, these findings suggest that inflammation-induced changes in enteric neurons and glial cells may contribute to negative affective states.

## 2 Discussion

Interoceptive signals from the gastrointestinal tract, conveyed through vagal pathways, spinal afferent pathways, ENS-related routes, and humoral/immune signaling, are increasingly recognized as important modulators of emotion-related and motivational states. Nevertheless, direct causal evidence linking these pathways to behavioral regulation remains limited, and rodent behavioral assays require careful interpretation. Many phenotypes described as anxiety-like, depression-like, aversion-related, or motivational may also reflect changes in arousal, sickness behavior, vigilance, metabolic state, visceral discomfort, pain, or locomotion. The studies reviewed here begin to narrow this gap by identifying candidate gut-derived signals and their associated cellular substrates. However, it remains unclear which specific molecular or cellular signals originating from the gut shape affective-like behaviors and how these signals are integrated into central circuits.

Although the ENS has been proposed as an important source of gut-derived signals, direct evidence linking ENS activity to emotion-related behavioral regulation remains limited. Multiple routes may connect the ENS and the central nervous system, including vagal pathways, spinal afferent pathways, epithelial intermediates, immune mediators, and humoral signaling. However, the relative contributions of these routes and how they are integrated to shape affective-like states remain to be clarified. Future studies should identify specific ENS-derived signals and dissect the neural circuits through which these signals are conveyed to and integrated in the brain. A comprehensive understanding of these mechanisms will be essential for clarifying how gastrointestinal physiology influences emotion-related behaviors and may provide new insights into the pathophysiology of psychiatric disorders.

Across these studies, several broader questions emerge. A key issue is whether distinct gut-to-brain pathways encode separable behavioral dimensions, such as negative valence, vigilance, reward, memory, or motivational drive. Gut-derived signals including nutrients, inflammatory mediators and microbiota-related factors may shape emotion-related and motivational behaviors through multiple routes. In particular, inflammatory signals may alter vagal and/or spinal afferent activity and increase visceral discomfort, thereby promoting or exacerbating anxiety- or depression-like behaviors.

An important future direction is to identify the molecular identities of these gut-derived signals and determine how they are integrated into central circuits. Future studies should combine gut-specific stimulation or inhibition with genetic manipulation of defined cell types and longitudinal activity recording. Careful behavioral controls will also be necessary to distinguish emotion-related changes from generalized physiological or motivational state changes.

## References

[ref1] Alvarez-Dieppa A. C. Griffin K. Cavalier S. McIntyre C. K. (2016). Vagus nerve stimulation enhances extinction of conditioned fear in rats and modulates arc protein, CaMKII, and GluN2B-containing NMDA receptors in the basolateral amygdala. Neural Plast. 2016:4273280. doi: 10.1155/2016/4273280, 27957346 PMC5120198

[ref2] Bai L. Mesgarzadeh S. Ramesh K. S. Huey E. L. Liu Y. Gray L. A. . (2019). Genetic identification of vagal sensory neurons that control feeding. Cell 179, 1129–1143.e23. doi: 10.1016/j.cell.2019.10.031, 31730854 PMC6916730

[ref3] Barberio B. Zamani M. Black C. J. Savarino E. V. Ford A. C. (2021). Prevalence of symptoms of anxiety and depression in patients with inflammatory bowel disease: a systematic review and meta-analysis. Lancet Gastroenterol. Hepatol. 6, 359–370. doi: 10.1016/S2468-1253(21)00014-5, 33721557

[ref4] Bercik P. Park A. J. Sinclair D. Khoshdel A. Lu J. Huang X. . (2011). The anxiolytic effect of *Bifidobacterium longum* NCC3001 involves vagal pathways for gut–brain communication. Neurogastroenterol. Motil. 23, 1132–1139. doi: 10.1111/j.1365-2982.2011.01796.x, 21988661 PMC3413724

[ref5] Biggio F. Gorini G. Utzeri C. Olla P. Marrosu F. Mocchetti I. . (2009). Chronic vagus nerve stimulation induces neuronal plasticity in the rat hippocampus. Int. J. Neuropsychopharmacol. 12, 1209–1221. doi: 10.1017/S1461145709000200, 19309534 PMC2879889

[ref6] Bisgaard T. H. Allin K. H. Keefer L. Ananthakrishnan A. N. Jess T. (2022). Depression and anxiety in inflammatory bowel disease: epidemiology, mechanisms and treatment. Nat. Rev. Gastroenterol. Hepatol. 19, 717–726. doi: 10.1038/s41575-022-00634-6, 35732730

[ref7] Bravo J. A. Forsythe P. Chew M. V. Escaravage E. Savignac H. M. Dinan T. G. . (2011). Ingestion of Lactobacillus strain regulates emotional behavior and central GABA receptor expression in a mouse via the vagus nerve. Proc. Natl. Acad. Sci. USA 108, 16050–16055. doi: 10.1073/pnas.1102999108, 21876150 PMC3179073

[ref8] Chen C. H. Tsai T. C. Wu Y. J. Hsu K. S. (2023). Gastric vagal afferent signaling to the basolateral amygdala mediates anxiety-like behaviors in experimental colitis mice. JCI Insight 8:e161874. doi: 10.1172/jci.insight.161874, 37200091 PMC10371239

[ref9] Conway C. R. Aaronson S. T. Sackeim H. A. George M. S. Zajecka J. Bunker M. T. . (2025). Vagus nerve stimulation in treatment-resistant depression: a one-year, randomized, sham-controlled trial. Brain Stimul. 18, 676–689. doi: 10.1016/j.brs.2024.12.1191, 39706521

[ref10] Cordner Z. A. Li Q. Liu L. Tamashiro K. L. Bhargava A. Moran T. H. . (2021). Vagal gut-brain signaling mediates amygdaloid plasticity, affect, and pain in a functional dyspepsia model. JCI Insight 6:e144046. doi: 10.1172/jci.insight.144046, 33591956 PMC8026195

[ref11] Diepenbroek C. Quinn D. Stephens R. Zollinger B. Anderson S. Pan A. . (2017). Validation and characterization of a novel method for selective vagal deafferentation of the gut. Am J Physiol 313, G342–G352. doi: 10.1152/ajpgi.00095.2017, 28705805 PMC5668568

[ref12] Han W. Tellez L. A. Perkins M. H. Perez I. O. Qu T. Ferreira J. . (2018). A neural circuit for gut-induced reward. Cell 175, 665–678.e23. doi: 10.1016/j.cell.2018.08.049, 30245012 PMC6195474

[ref13] Hwang J. Lee S. Okada J. Liu L. Pessin J. E. Chua S. C. . (2025). Liver-innervating vagal sensory neurons are indispensable for the development of hepatic steatosis and anxiety-like behavior in diet-induced obese mice. Nat. Commun. 16:991. doi: 10.1038/s41467-025-56328-5, 39856118 PMC11759694

[ref14] Ichiki T. Wang T. Kennedy A. Pool A. H. Ebisu H. Anderson D. J. . (2022). Sensory representation and detection mechanisms of gut osmolality change. Nature 602, 468–474. doi: 10.1038/s41586-021-04359-5, 35082448

[ref15] Klarer M. Arnold M. Günther L. Winter C. Langhans W. Meyer U. (2014). Gut vagal afferents differentially modulate innate anxiety and learned fear. J. Neurosci. 34, 7067–7076. doi: 10.1523/JNEUROSCI.0252-14.2014, 24849343 PMC6608191

[ref16] Krahl S. E. Senanayake S. S. Pekary A. E. Sattin A. (2004). Vagus nerve stimulation (VNS) is effective in a rat model of antidepressant action. J. Psychiatr. Res. 38, 237–240. doi: 10.1016/j.jpsychires.2003.11.005, 15003428

[ref17] Krieger J. P. Asker M. van der Velden P. Börchers S. Richard J. E. Maric I. . (2022). Neural pathway for gut feelings: vagal interoceptive feedback from the gastrointestinal tract is a critical modulator of anxiety-like behavior. Biol. Psychiatry 92, 709–721. doi: 10.1016/j.biopsych.2022.04.020, 35965105 PMC11438499

[ref18] Kupari J. Häring M. Agirre E. Castelo-Branco G. Ernfors P. (2019). An atlas of vagal sensory neurons and their molecular specialization. Cell Rep. 27, 2508–2523.e4. doi: 10.1016/j.celrep.2019.04.096, 31116992 PMC6533201

[ref19] Li Y. Wang Y. Sun Q. Li M. Y. Xu J. Z. Li Y. Q. . (2024). Inhibiting the activation of enteric glial cells alleviates intestinal inflammation and comorbid anxiety- and depressive-like behaviors in the ulcerative colitis mice. Neurochem. Int. 178:105789. doi: 10.1016/j.neuint.2024.105789, 38852824

[ref20] McGuinness A. J. Davis J. A. Dawson S. L. Loughman A. Collier F. O’Hely M. . (2022). A systematic review of gut microbiota composition in observational studies of major depressive disorder, bipolar disorder and schizophrenia. Mol. Psychiatry 27, 1920–1935. doi: 10.1038/s41380-022-01456-3, 35194166 PMC9126816

[ref21] Millett C. J. Shaver J. J. Bracken B. Jones S. J. Lovelett R. J. Rubinow D. A. . (2025). In vivo transcriptomic, functional, circuit-based, and translational analyses of enteric neurons. Cell 188, 7547–7570.e45. doi: 10.1016/j.cell.2025.11.024, 41406962

[ref22] Noble L. J. Gonzalez I. J. Meruva V. B. Callahan K. A. Belfort B. D. Ramanathan K. R. . (2017). Effects of vagus nerve stimulation on extinction of conditioned fear and post-traumatic stress disorder symptoms in rats. Transl. Psychiatry 7:e1217. doi: 10.1038/tp.2017.191, 28892066 PMC5611754

[ref23] Noble L. J. Meruva V. B. Hays S. A. Rennaker R. L. Kilgard M. P. McIntyre C. K. (2019). Vagus nerve stimulation promotes generalization of conditioned fear extinction and reduces anxiety in rats. Brain Stimulat. 12, 9–18. doi: 10.1016/j.brs.2018.09.013, 30287193 PMC6301121

[ref24] Okonogi T. Kuga N. Yamakawa M. Kayama T. Ikegaya Y. Sasaki T. (2024). Stress-induced vagal activity influences anxiety-relevant prefrontal and amygdala neuronal oscillations in male mice. Nat. Commun. 15:183. doi: 10.1038/s41467-023-44205-y, 38195621 PMC10776769

[ref25] Onimus O. Arrivet F. Le Borgne T. Perez S. Castel J. Ansoult A. . (2026). The gut-brain vagal axis governs mesolimbic dopamine dynamics and reward events. Sci. Adv. 12:eadz0828. doi: 10.1126/sciadv.adz0828, 41616060 PMC12857734

[ref26] Peña D. F. Childs J. E. Willett S. Vital A. McIntyre C. K. Kroener S. (2014). Vagus nerve stimulation enhances extinction of conditioned fear and modulates plasticity in the pathway from the ventromedial prefrontal cortex to the amygdala. Front. Behav. Neurosci. 8:327. doi: 10.3389/fnbeh.2014.00327, 25278857 PMC4166996

[ref27] Peña D. F. Engineer N. D. McIntyre C. K. (2013). Rapid remission of conditioned fear expression with extinction training paired with Vagus nerve stimulation. Biol. Psychiatry 73, 1071–1077. doi: 10.1016/j.biopsych.2012.10.021, 23245749 PMC3604026

[ref28] Sahasrabudhe A. Rupprecht L. E. Orguc S. Khudiyev T. Tanaka T. Sands J. . (2024). Multifunctional microelectronic fibers enable wireless modulation of gut and brain neural circuits. Nat. Biotechnol. 42, 892–904. doi: 10.1038/s41587-023-01833-5, 37349522 PMC11180606

[ref29] Scott K. A. Tan Y. Johnson D. N. Elsaafien K. Baumer-Harrison C. Méndez-Hernández R. . (2025). Mechanosensation of the heart and gut elicits hypometabolism and vigilance in mice. Nat. Metab. 7, 263–275. doi: 10.1038/s42255-024-01205-6, 39824919 PMC12232619

[ref30] Shi D. Reddy P. Marrin M. Walker C. Siu C. Muller P. A. . (2025). Properties and functions of transcriptionally distinct enteric neurons. Cell 188, 7571–7590.e35. doi: 10.1016/j.cell.2025.10.041, 41297538

[ref31] Siopi E. Galerne M. Rivagorda M. Saha S. Moigneu C. Moriceau S. . (2023). Gut microbiota changes require vagus nerve integrity to promote depressive-like behaviors in mice. Mol. Psychiatry 28, 3002–3012. doi: 10.1038/s41380-023-02071-6, 37131071 PMC10615761

[ref32] Souza R. R. Robertson N. M. Mathew E. Tabet M. N. Bucksot J. E. Pruitt D. T. . (2020). Efficient parameters of vagus nerve stimulation to enhance extinction learning in an extinction-resistant rat model of PTSD. Prog. Neuro-Psychopharmacol. Biol. Psychiatry 99:109848. doi: 10.1016/j.pnpbp.2019.109848, 31863872

[ref33] Souza R. R. Robertson N. M. McIntyre C. K. Rennaker R. L. Hays S. A. Kilgard M. P. (2021). Vagus nerve stimulation enhances fear extinction as an inverted-U function of stimulation intensity. Exp. Neurol. 341:113718. doi: 10.1016/j.expneurol.2021.113718, 33844986

[ref34] Wang T. T. Luo Y. J. Feng W. Jiang L. X. Yu Q. S. He Y. T. . (2026). Gut microbial metabolism via hippocampal indole-AhR signaling regulates emotional symptoms. Cell Metab. doi: 10.1016/j.cmet.2026.03.003, 41928504

[ref001] Wang T. Tufenkjian A. Ajijola O. A. Oka Y. (2025). Molecular and functional diversity of the autonomic nervous system. Nat. Rev. Neurosci 26, 607–622. doi: 10.1038/s41583-025-00941-2, 40610604

[ref35] Wang Z. Tang Q. Li K. Mou J. Chen Y. Kuang W. . (2026). An enteric-DRG pathway for interoception and visceral pain in mice. Neuron 114, 105–121.e6. doi: 10.1016/j.neuron.2025.09.035, 41135523

[ref36] Yang M. Singh A. de Araujo A. McDougle M. Ellis H. Décarie-Spain L. . (2025). Separate orexigenic hippocampal ensembles shape dietary choice by enhancing contextual memory and motivation. Nat. Metab. 7, 276–296. doi: 10.1038/s42255-024-01194-6, 39815079 PMC11860247

